# Dipeptidyl peptidase-4 inhibitor anagliptin reduces fasting apolipoprotein B-48 levels in patients with type 2 diabetes: A randomized controlled trial

**DOI:** 10.1371/journal.pone.0228004

**Published:** 2020-01-28

**Authors:** Takeshi Onoue, Motomitsu Goto, Eri Wada, Mariko Furukawa, Takayuki Okuji, Norio Okada, Tomoko Kobayashi, Shintaro Iwama, Mariko Sugiyama, Taku Tsunekawa, Hiroshi Takagi, Daisuke Hagiwara, Yoshihiro Ito, Yoshiaki Morishita, Yusuke Seino, Hidetaka Suga, Ryoichi Banno, Yoji Hamada, Masahiko Ando, Etsuko Yamamori, Hiroshi Arima

**Affiliations:** 1 Department of Endocrinology and Diabetes, Nagoya University Graduate School of Medicine, Nagoya, Japan; 2 Research Center of Health, Physical Fitness and Sports, Nagoya University, Nagoya, Japan; 3 Center for Advanced Medicine and Clinical Research, Nagoya University Hospital, Nagoya, Japan; 4 Kainan Hospital, Aichi Prefectural Welfare Federation of Agricultural Cooperatives, Aichi, Japan; Universidad Miguel Hernandez de Elche, SPAIN

## Abstract

Type 2 diabetes and dyslipidemia are diseases that collectively increase the risk of patients developing cardiovascular complications. Several incretin-based drugs are reported to improve lipid metabolism, and one of these medications, anagliptin, is a dipeptidyl peptidase-4 (DPP-4) inhibitor that has been shown to decrease serum triglyceride and low-density lipoproteins cholesterol. This study aimed to conduct an investigation into the effects of anagliptin on serum lipid profiles. This multicenter, open-label, randomized (1:1), parallel group study was designed to evaluate the effects of anagliptin on serum lipid profiles (triglycerides, lipoproteins, apolipoproteins, and cholesterol fractions). The study involved 24 patients with type 2 diabetes at two participating hospitals for a period of 24 weeks. Patients were randomly assigned to the anagliptin (n = 12) or control (n = 12) groups. Patients in the anagliptin group were treated with 200 mg of the drug twice daily. Patients in the control group did not receive anagliptin, but continued with their previous treatment schedules. Lipid metabolism was examined under fasting conditions at baseline and 24 weeks. Patients treated with anagliptin for 24 weeks exhibited significantly reduced levels of serum apolipoprotein B-48, a marker for lipid transport from the intestine, compared with the control group patients (P < 0.05). After 24 weeks of treatment, serum adiponectin levels were significantly raised, whereas glycated hemoglobin (HbA1c) levels were significantly lower compared with the baseline in the anagliptin group (P < 0.05), but not in the control group. This study showed that the DPP-4 inhibitor anagliptin reduces fasting apolipoprotein B-48 levels, suggesting that this drug may have beneficial effects on lipid metabolism possibly mediated by the inhibition of intestinal lipid transport.

## Introduction

Type 2 diabetes is accompanied frequently by various abnormalities of lipid metabolism including high levels of triglycerides and low-density lipoproteins (LDL) and low levels of high-density lipoproteins (HDL) [1]. Type 2 diabetes and dyslipidemia are reported collectively to increase the risks of developing cardiovascular disease [2].

Dipeptidyl peptidase-4 (DPP-4) inhibitors are a class of oral hypoglycemic agents that increase incretins such as glucagon-like peptide 1 (GLP-1) and glucose-dependent insulinotropic polypeptide, and they have been used widely in the treatment of type 2 diabetes. In addition to their hypoglycemic effects, DPP-4 inhibitors have been reported to have protective effects on pancreatic β-cells [3, 4], anti-atherogenic effects [5–7], renoprotective effects [8, 9], and favorable effects on lipid metabolism [10–12].

Anagliptin, a DPP-4 inhibitor launched in 2012, reportedly lowered LDL cholesterol levels in a long-term phase III trial [13] and, since then, several studies have confirmed its beneficial effect on lipid metabolism by showing that the drug decreases serum cholesterol and triglyceride levels in patients with type 2 diabetes [14, 15]. A recent study further reported that anagliptin significantly lowered LDL cholesterol compared with sitagliptin [16]. However, the effects of anagliptin on lipid profiles have not fully been investigated.

The present randomized controlled trial was carried out to evaluate the effects of 24 weeks of anagliptin treatment on serum lipid profiles (triglycerides, lipoproteins, apolipoproteins, and cholesterol fractions) in patients with type 2 diabetes complicated by dyslipidemia. The findings will help to increase our understanding of the effects that treating patients with anagliptin has on lipid metabolism.

## Materials and methods

### Trial design

This multicenter, open-label, randomized (1:1), parallel group study was conducted in patients with type 2 diabetes at two participating hospitals: Nagoya University Hospital and Kainan Hospital. The study protocol was approved by the Ethical Committee of Nagoya University Graduate School of Medicine (No. 2013–0266). This clinical trial was listed in the Japanese University Hospital Medical Information Network Clinical Trials Registry (UMIN-CTR: UMIN000014531 URL: https://upload.umin.ac.jp/cgi-open-bin/ctr_e/ctr_view.cgi?recptno=R000014762). All enrolled patients provided written consent to participate after they were informed of the purpose of the study as well as the potential risks and benefits. This study was performed in accordance with the ethical principles of the Declaration of Helsinki and the Ethical Guidelines for Medical and Health Research Involving Human Subjects.

### Patients

Patients were eligible for inclusion if they 1) had type 2 diabetes, 2) had glycosylated hemoglobin (HbA1c) ≥ 6.5%, 3) had serum LDL cholesterol ≥ 120 mg/dl, 4) had not been treated with DPP-4 inhibitors for the previous 3 months, 5) took no antidiabetic medication or had not changed their medication for the previous 3 months, 6) had less than 2% HbA1c change for the past 3 months, and 7) were 20 years-of-age or older. Patients were excluded if they 1) were treated with insulin, glinides or high doses of sulfonylureas (glimepiride > 2 mg/day, gliclazide > 40 mg/day, glibenclamide > 1.25 mg/day), 2) had hypoglycemia unawareness or recurrent severe hypoglycemia, 3) had severe forms of liver disease (aspartate transaminase or alanine aminotransferase ≥ 100 IU/L), 4) were on dialysis, 5) had severe forms of cardiac disease, 6) had severe anemia (Hb 8.0 < g/dl), 7) had severe pancreatitis, 8) had any malignant diseases, 9) had uncontrolled endocrine diseases, 9) had severe infections or were undergoing surgical treatment, 10) had severe diabetic complications, 11) had inflammatory bowel disease, colon ulcers, ileus, or a history of intestinal resection, 12) had an addiction to alcohol, 13) were or possibly were pregnant, 14) had an allergy to DPP-4 inhibitors, or 15) were judged by their physicians to be unsuitable for participation in the study.

Because this study was exploratory, it was difficult to estimate changes in lipid profiles. A previous study indicated that (mean difference between groups/standard deviation [SD]) could represent the effect size and reported the medium and large effect sizes as 0.5 and 0.8, respectively [17]. Therefore, we planned to recruit 60 participants (30 in each group) to detect the difference between the groups with a P-value of 0.05 (two sided) and a statistical power of 70%–80% based on the assumption that the effect size would be 0.65–0.80. Participants were recruited from patients meeting the above criteria who visited one of the two participating hospitals during the period from July 2014 to November 2017.

### Registration and randomization

After consent was obtained from the patients, the researcher accessed a web-based registration system developed by the Center for Advanced Medical and Clinical Research at Nagoya University Hospital and entered the information required for enrollment. The system automatically determined the eligibility of each patient and randomly assigned him/her to the anagliptin or control group (1:1) with a dynamic allocation strategy using a minimization method. Stratification included the hospital that the patient visited, sex, and age (> 55 or ≤ 55).

### Interventions

Patients in the anagliptin group were treated with 200 mg per day of the drug twice daily for 24 weeks. Patients in the control group did not receive anagliptin but continued with their previous treatment schedules. Throughout the study, no medications were added but those drugs that the patients were taken at enrollment continued to be administered. Lipid metabolism markers were measured under fasting conditions at baseline and 24 weeks.

### Outcomes

The primary outcome was the occurrence of changes in serum lipid profiles (triglycerides, lipoproteins, apolipoproteins, and cholesterol fraction) between baseline and 24 weeks after initiation of treatment with anagliptin. Secondary outcomes included changes in body weight, body mass index, blood pressure, glycometabolism (HbA1c, fasting plasma glucose, insulin, and homeostasis model assessment of insulin resistance [18]), cytokine levels [serum adiponectin, serum tumor necrosis factor (TNF) α, and serum monocyte chemoattractant protein (MCP) 1], and levels of an oxidative stress marker [serum malondialdehyde-modified (MDA)-LDL] and an inflammation marker [serum high sensitive C-reactive protein (CRP)].

### Laboratory tests

Measurements of serum lipid profiles and other parameters were outsourced to a commercial clinical testing laboratory (SRL, Tokyo, Japan) and analyzed as described previously [14, 19, 20].

### Statistical analysis

Continuous variables were expressed as means (SD). Between-group differences in baseline values of continuous variables were tested for significance with the two-sample *t*-test and values of nominal variables were compared using Fisher’s exact test. Changes in parameters between baseline and 24 weeks were evaluated in each group and compared using an analysis of covariance (ANCOVA) model including baseline value and treatment group as covariates. The permutation test was also performed to compare the changes in serum lipid profiles between groups. Because this study was exploratory, no adjustment for multiplicity was performed and analyses were conducted using two-sided tests at a significance level of 0.05. SAS 9.4 software from the SAS Institute Inc., Cary, NC, USA, was used for all calculations.

## Results

**Fig 1:**
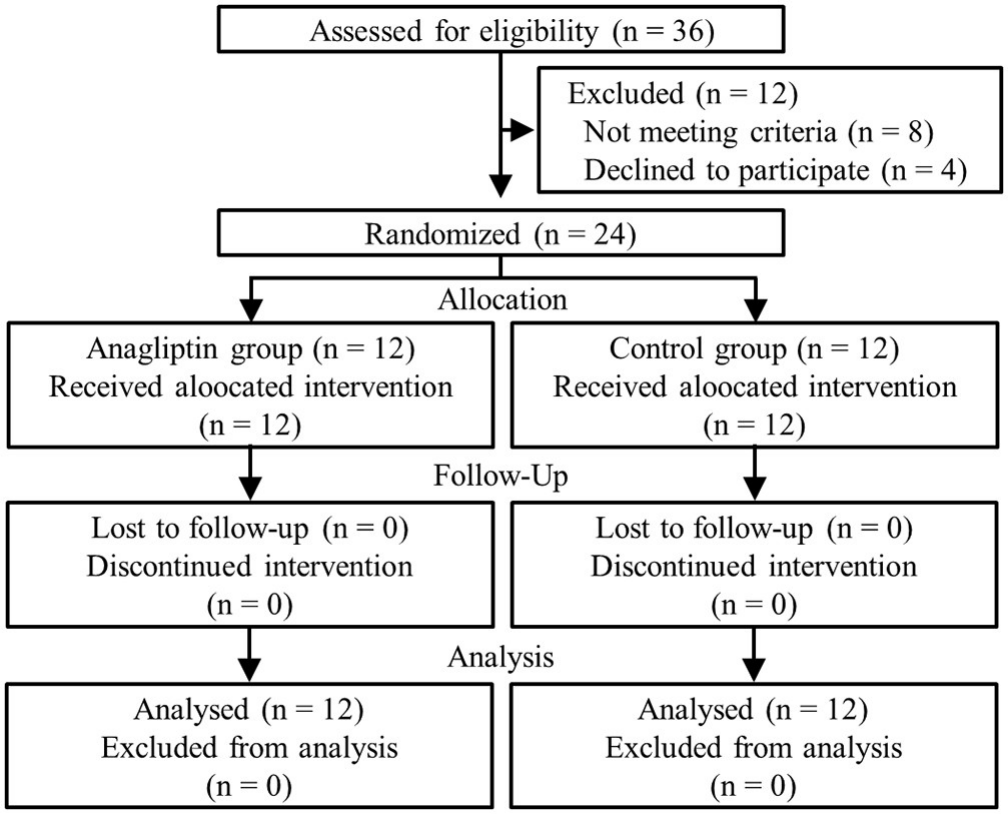
Study flowchart. Overall, 24 patients were randomly assigned to the anagliptin (n = 12) or control (n = 12) group. Patients in the anagliptin group received 200 mg per day of the drug twice daily for 24 weeks, whereas those in the control group did not receive anagliptin but continued with their previous treatment schedules.

Because DPP-4 inhibitors were widely used in the participating hospitals, 36 candidates were eligible for this study at the initial screening, based on electronic medical records. Of the 36 patients, 8 did not meet the eligibility criteria because of the patients’ declaration status (3 had malignant diseases, 2 had severe forms of cardiac disease, 2 had severe forms of liver disease, and 1 was undergoing surgical treatment) and 4 patients refused to enroll in the study because they were not interested in participating in the research. Therefore, 24 patients were recruited into the study, and were randomly assigned to the anagliptin (n = 12) or control (n = 12) group. A study flowchart is shown in Fig 1.

Baseline characteristics are displayed in Table 1. The mean age, body mass index, and HbA1c levels of the patients were 63.9 ± 13.3 years, 26.2 ± 4.9 kg/m^2^, and 7.2% ± 0.5%, respectively. No between-group differences in baseline characteristics were observed, except for triglycerides. The data on hypoglycemic drugs and lipid-lowering drugs taken by patients in both groups are shown in S1 Table. There were no significant differences in the treatments between the two groups. There were no severe adverse events related to interventions.

**Table 1:** Baseline characteristics of the study population.

	Total (n = 24)	Anagliptin group (n = 12)	Control group (n = 12)	P-value
Age (years)	63.9 (13.3)	64.8 (12.4)	62.9 (14.7)	0.73
Sex				
Female	15	7	8	1.00
Male	9	5	4	0.19
Duration of diabetes (years)	7.6 (6.6)	7.7 (4.6)	7.4 (8.3)	
Body weight (kg)	70.4 (15.6)	71.4 (18.8)	69.3 (12.3)	0.75
Body mass index (kg/m^2^)	26.2 (4.9)	26.9 (6.0)	25.6 (3.5)	0.52
Blood pressure (mmHg)				
Systolic blood pressure	128 (14)	129 (15)	127 (13)	0.77
Diastolic blood pressure	73 (7)	73 (8)	72 (7)	0.66
HbA1c (%)	7.2 (0.5)	7.1 (0.5)	7.3 (0.5)	0.41
Plasma glucose (mg/dl)	147 (21)	143 (20)	152 (23)	0.36
Total cholesterol (mg/dl)	223 (29)	224 (30)	222 (30)	0.87
Triglyceride (mg/dl)	140 (57)	166 (64)	113 (35)	**0.02**
HDL cholesterol (mg/dl)	50 (11)	50 (10)	49 (13)	0.80
LDL cholesterol (mg/dl)	151 (22)	149 (21)	152 (24)	0.69
Use of hypoglycemic drugs	24 (100%)	12 (100%)	12 (100%)	1.00
Use of antihypertensive drugs	8 (33%)	5 (42%)	3 (25%)	0.67
Use of lipid-lowering drugs	11 (46%)	4 (33%)	7 (58%)	0.41

Data are expressed as mean (SD) or n (%). HDL, high-density lipoprotein; LDL, low-density lipoprotein. P-values < 0.05 are shown in bold.

The primary outcomes, changes in serum lipid profiles (triglycerides, lipoproteins, apolipoproteins, and cholesterol fraction) between baseline and 24 weeks, are shown in Table 2. Treatment with anagliptin for 24 weeks significantly reduced fasting serum apolipoprotein (Apo) B-48 compared with the control group (P < 0.05). Fasting serum triglyceride levels were significantly lower at 24 weeks compared with the baseline in both groups. Serum remnant lipoprotein (RLP) cholesterol levels were significantly lower at 24 weeks compared with the baseline in the control group, but not in the anagliptin group. There were no significant changes in the levels of total, HDL, and LDL cholesterols, Apo A-I, total Apo B, Apo E, and cholesterol fractions in both groups. We also performed a permutation test and confirmed that the treatment with anagliptin significantly reduced fasting serum Apo B-48 compared with the control group (P = 0.013) (S2 Table). In an analysis that excluded patients taking statins, the treatment with anagliptin significantly reduced fasting serum Apo B-48 compared with the control group (data not shown).

**Table 2:** Changes in serum lipid profiles between baseline and 24 weeks.

	Anagliptin group (n = 12) (SD)			Control group (n = 12) (SD)			Difference between groups P-value
	baseline	24 weeks	24 weeks vs baseline P-value	baseline	24 weeks	24 weeks vs. baseline P-value	
Total cholesterol (mg/dl)	224 (30)	225 (35)	0.88	222 (30)	218 (29)	0.57	0.61
Triglyceride (mg/dl)	166 (64)	131 (41)	**0.0499**	113 (35)	98 (39)	**0.01**	0.58
HDL cholesterol (mg/dl)	50 (10)	52 (12)	0.42	49 (13)	51 (13)	0.42	1.00
LDL cholesterol (mg/dl)	149 (21)	152 (30)	0.73	152 (24)	147 (20)	0.58	0.52
RLP cholesterol (mg/dl)	6.8 (2.9)	6.0 (1.6)	0.30	6.2 (2.4)	4.9 (3.1)	**0.02**	0.35
Apo A-I (mg/dl)	145 (22)	146 (27)	0.70	136 (27)	139 (25)	0.76	0.95
Apo B (mg/dl)	118 (21)	114 (21)	0.48	114 (16)	110 (16)	0.29	0.80
Apo B-48 (μg/ml)	5.8 (5.9)	4.1 (3.6)	**0.04**	4.1 (2.4)	5.3 (3.3)	0.19	**0.02**
Apo E (mg/dl)	4.3 (1.1)	4.1 (0.9)	0.30	4.4 (0.9)	4.2 (1.2)	0.31	0.99
Lathosterol (μg/ml)	3.1 (1.4)	2.9 (1.7)	0.86	2.5 (1.1)	2.9 (1.5)	0.49	0.54
Campesterol (μg/ml)	4.1 (1.4)	4.6 (1.8)	0.83	5.5 (1.7)	5.9 (2.3)	0.22	0.49
Sitosterol (μg/ml)	2.0 (0.6)	2.4 (0.9)	0.58	3.1 (1.1)	3.4 (1.2)	0.07	0.40

Change in parameters between baseline and 24 weeks were evaluated in each group and compared using an analysis of covariance (ANCOVA) model including the baseline value and treatment group as covariates. SD, standard deviation; HDL, high-density lipoprotein; LDL, low-density lipoprotein; RLP, remnant lipoprotein; Apo, apolipoprotein. P-values < 0.05 are shown in bold.

Changes in body weight, body mass index, blood pressure, glycometabolism, serum cytokines, serum MDA-LDL, and serum high sensitive CRP between baseline and 24 weeks are shown in Table 3. Serum adiponectin levels were significantly higher at 24 weeks compared with baseline in the anagliptin group (7.4 ± 3.7 μg/ml to 8.6 ± 3.9 μg/ml, P < 0.05), but not in the control group. HbA1c levels were significantly lower at 24 weeks compared with baseline in the anagliptin group (7.1% ± 0.5% to 6.8% ± 0.6%, P < 0.05), but not in the control group, although there was no statistically significant difference in the changes between baseline and 24 weeks between groups. There were no significant changes between both groups in body weight, body mass index, systolic blood pressure, diastolic blood pressure, fasting plasma glucose, serum insulin, homeostasis model assessment of insulin resistance, serum TNF α, serum MCP-1, serum MDA-LDL, and serum high sensitive CRP levels.

**Table 3:** Changes in glycometabolism, cytokines, oxidative stress markers, and inflammation marker between baseline and 24 weeks in patients receiving anagliptin.

	Anagliptin group (n = 12) (SD)			Control group (n = 12) (SD)			Difference between groups P-value
	baseline	24 weeks	24 weeks vs baseline P-value	baseline	24 weeks	24 weeks vs baseline P-value	
Body weight (kg)	71.4 (18.8)	70.2 (18.4)	0.08	69.3 (12.3)	69.3 (12.0)	0.97	0.22
Body mass index (kg/m^2^)	26.9 (6.0)	26.4 (5.9)	0.09	25.6 (3.5)	25.6 (3.3)	0.94	0.25
SBP (mmHg)	129 (15)	130 (9)	0.79	127 (13)	130 (20)	0.62	0.87
DBP (mmHg)	73 (8)	74 (11)	0.91	72 (7)	73 (11)	0.56	0.74
HbA1c (%)	7.1 (0.5)	6.8 (0.6)	**0.04**	7.3 (0.5)	7.1 (0.6)	0.46	0.31
Plasma glucose (mg/dl)	143 (20)	131 (26)	0.21	152 (23)	155 (48)	0.75	0.27
Insulin (μIU/ml)	9.1 (6.5)	6.5 (3.9)	0.25	9.5 (9.2)	10.7 (11.3)	0.57	0.22
HOMA-IR	3.2 (2.0)	2.1 (1.2)	0.38	3.6 (3.4)	4.9 (6.7)	0.30	0.18
Adiponectin (μg/ml)	7.4 (3.7)	8.6 (3.9)	**0.01**	8.7 (4.5)	8.9 (4.7)	0.50	0.11
TNFα (pg/ml)	1.27 (0.45)	1.26 (0.30)	0.60	1.11 (0.38)	1.11 (0.31)	0.49	0.40
MCP-1 (pg/ml)	268 (46)	255 (54)	0.27	289 (83)	284 (76)	0.93	0.47
MDA-LDL (U/l)	174 (49)	168 (41)	0.70	162 (35)	152 (40)	0.20	0.52
High sensitive CRP (mg/l)	1.2 (1.1)	0.8 (0.7)	0.47	1.9 (2.5)	2.1 (3.9)	0.62	0.40

Change in parameters between baseline and 24 weeks were evaluated for each group and compared using an analysis of covariance (ANCOVA) model including the baseline value and treatment group as covariates. SD, standard deviation; SBP, systolic blood pressure; DBP, diastolic blood pressure; HOMA-IR, homeostasis model assessment of insulin resistance; TNFα, tumor necrosis factor α; MCP-1, monocyte chemoattractant protein 1; MDA, malondialdehyde-modified; LDL, low-density lipoprotein; CRP, C-reactive protein. P-values < 0.05 are shown in bold.

## Discussion

In this randomized controlled study, we showed that treatment of patients with anagliptin for 24 weeks lead to significant reductions in fasting serum Apo B-48 levels, suggesting that the beneficial effects of anagliptin on lipid metabolism might be mediated by the inhibition of intestinal lipid transport.

Apo B is an apolipoprotein that composes TG-rich lipoproteins. Apo B-48 is synthesized in the intestine and Apo B-100 is synthesized in the liver. Apo B-48 is involved in the transport of TG-rich lipoproteins from the intestine and Apo B-100 is involved mainly in liver-based synthesis of very low-density lipoproteins. Apo B-48 forms chylomicron complex in intestinal epithelial cells, and the complex is transported from the basement membrane side of the intestine to the blood via lymphatic channels. In Apopec 1 knockout mice, in which Apo B-48 is not produced, intestinal Apo B secretion is reduced, leading to reduced transport of TG-rich lipoproteins [21]. In humans, Apo B-48 has been shown to have a significant positive correlation with total cholesterol and triglyceride levels, but a negative correlation with HDL cholesterol levels [22]. Therefore, Apo B-48 is considered to be a marker for both lipid transport from the intestine and postprandial hyperlipidemia [23].

Previous studies on mice have shown that blocking endogenous GLP-1 signaling cascades enhances the intestinal secretion of Apo B-48 [24] and treatment with anagliptin lowers serum total and non-HDL cholesterol levels by suppressing intestinal cholesterol transport mechanisms [25]. Published reports have also demonstrated that treatment with exenatide, a GLP-1 receptor agonist, suppresses the plasma concentration and production rate of Apo B-48, but not of Apo B-100, in healthy humans [26] and DPP-4 inhibitors such as vildagliptin, sitagliptin, and alogliptin lower Apo B-48 concentrations in patients with type 2 diabetes [10, 11, 27, 28]. As for the effects of anagliptin on lipid metabolism in humans, a single-arm study showing that 24 weeks of anagliptin treatment decreased postprandial, but not fasting, Apo B-48 levels. The reason for the discrepancy between that study and ours is not clear, but both suggest anagliptin has beneficial effects on lipid metabolism.

Although anagliptin treatment reportedly lowers LDL cholesterol levels, the changes are relatively small (3.0%–6.4%) [13–15]. Furthermore, a previous study showed that although the lowering effects of anagliptin on LDL cholesterol levels were observed in patients with baseline LDL cholesterol level of ≥ 140 mg/dl, they were not significant in patients with baseline LDL cholesterol level of ≥ 120 mg/dl [14]. Our criterion for baseline LDL cholesterol values was ≥ 120 mg/dl; we believe that we did not observe the lowering effects of anagliptin on LDL cholesterol owing to our baseline criterion. Nonetheless, we observed a significant decrease in Apo B-48 by anagliptin in the present study, indicating the importance of Apo B-48 regulation by the GLP-1 receptor signal, which has been suggested in previous studies [24, 26]. In addition, several studies with multivariate analyses have shown that fasting Apo B-48 was elevated independently of fasting plasma lipids in patients with coronary artery disease, those with stroke, and those with type 2 diabetes with asymptomatic peripheral arterial disease [29–31]. Moreover, Apo B-48 reportedly binds to arterial wall proteoglycans [32] and is found in human atherosclerotic plaques from femoral and carotid endarterectomy samples [33], suggesting that Apo B-48 is a marker for atherosclerotic disease as well as a potential therapeutic target.

Our data are consistent with those of a previous study [34] in that they show that anagliptin treatment increases serum adiponectin levels over the course of 24 weeks. Patients with type 2 diabetes have also been reported to show postprandial decreases in adiponectin levels that were inversely correlated with the levels of TG-rich lipoprotein [35]. Adiponectin was shown to be an independent predictor of plasma Apo B-48 in a multivariate regression analysis, [36] and therefore, the increased adiponectin levels may have contributed to the lowered levels of Apo B-48 seen in this study.

There are limitations in this study. First, the sample size is small. This may explain why there were no significant differences in HbA1c and other lipid profiles than Apo B-48 between groups. Nevertheless, we could detect a significant difference in Apo B-48 levels, probably owing to its relatively large effect size (mean difference between groups/SD = 0.94), highlighting the importance of the regulation of Apo B-48 by DPP-4 inhibitors. Second, because this study was exploratory, adjustment for multiplicity was not performed. Further studies for more specific outcomes should be performed in future based on the results of this study.

## Conclusions

In conclusion, this study indicates that the DPP-4 inhibitor anagliptin reduces fasting levels of Apo B-48, suggesting that the improvements in lipid metabolism induced by anagliptin may be due to the inhibition of intestinal lipid transport, and anagliptin has potential benefits for patients with diabetes and dyslipidemia.

## Supporting information

S1 Table Data on hypoglycemic drugs, antihypertensive drugs, and lipid-lowering drugs taken by patients in both groups. Throughout the study, no medications were added but the drugs taken by patients at enrollment continued to be administered. Data are expressed as n (%). SGLT2i, sodium glucose transporter-2 inhibitor. P-values <0.05 are shown in bold.
(DOCX)

S2 Table Changes in serum lipid profiles between baseline and 24 weeks analyzed using a permutation test. Change in parameters between baseline and 24 weeks were also compared using a permutation test. HDL, high-density lipoprotein; LDL, low-density lipoprotein; RLP, remnant lipoprotein; Apo, apolipoprotein. P-values <0.05 are shown in bold.
(DOCX)

S1 Appendix CONSORT checklist. (PDF)

S2 Appendix Study protocol (Japanese). (PDF)

S3 Appendix Study protocol (English). (PDF)

S4 Appendix Data. (XLSX)
