# Supplementary material for: Dipeptidyl peptidase-4 inhibitor anagliptin reduces fasting apolipoprotein B-48 levels in patients with type 2 diabetes: A randomized controlled trial
Source: PLoS One. 2020 Jan 28;15(1):e0228004. doi: 10.1371/journal.pone.0228004 (PMC6986701; doi:10.1371/journal.pone.0228004)
Supplement: S1 Table — Throughout the study, no medications were added but the drugs taken by patients at enrollment continued to be administered. Data are expressed as n (%). SGLT2i, sodium glucose transporter-2 inhibitor. P-values <0.05 are shown in bold. (DOCX) [file pone.0228004.s001.docx]

**S1 Table. The information on hypoglycemic drugs, antihypertensive drug, and lipid-lowering drugs that patients in both groups.**

|  | Anagliptin  group (n = 12) | Control  group (n = 12) | P-value |
| --- | --- | --- | --- |
| **Use of hypoglycemic drugs** |  |  |  |
| Metformin | 4 (33%) | 7 (58%) | 0.41 |
| Sulfonylurea | 2 (17%) | 4 (33%) | 0.64 |
| SGLT2i | 1 (8%) | 1 (8%) | 1.00 |
| Thiazolidine | 1 (8%) | 1 (8%) | 1.00 |
| α-glucosidase inhibitor | 4 (33%) | 6 (50%) | 0.68 |
| Glinide | 2 (17%) | 2 (17%) | 1.00 |
| Insulin | 2 (17%) | 2 (17%) | 1.00 |
| **Use of antihypertensive drugs** |  |  |  |
| Calcium channel blocker | 3 (25%) | 2 (17%) | 1.00 |
| Angiotensin II receptor blocker | 4 (33%) | 3 (25%) | 1.00 |
| Diuretic | 1 (8%) | 1 (8%) | 1.00 |
| **Use of lipid-lowering drugs** |  |  |  |
| Statin | 1 (8%) | 3 (25%) | 0.59 |
| Fibrate | 1 (8%) | 1 (8%) | 1.00 |
| Ezetimibe | 1 (8%) | 1 (8%) | 1.00 |
| Ethyl icosapentate | 1 (8%) | 2 (17%) | 1.00 |

Throughout the study, no medications were added but those drugs that the patients were taken at enrollment continued to be administered. Data are expressed as n (%). SGLT2i, Sodium glucose transporter-2 inhibitor. P-values < 0.05 are shown in bold.
