# Supplementary material for: Dipeptidyl peptidase-4 inhibitor anagliptin reduces fasting apolipoprotein B-48 levels in patients with type 2 diabetes: A randomized controlled trial
Source: PLoS One. 2020 Jan 28;15(1):e0228004. doi: 10.1371/journal.pone.0228004 (PMC6986701; doi:10.1371/journal.pone.0228004)
Supplement: S2 Table — Change in parameters between baseline and 24 weeks were also compared using a permutation test. HDL, high-density lipoprotein; LDL, low-density lipoprotein; RLP, remnant lipoprotein; Apo, apolipoprotein. P-values <0.05 are shown in bold. (DOCX) [file pone.0228004.s002.docx]

**S2 Table.** **Changes in serum lipid profiles between baseline and 24 weeks analyzed using a permutation test.**

|  | Permutation p-value |
| --- | --- |
|  |  |
| Total cholesterol (mg/dl) | 0.69 |
| Triglyceride (mg/dl) | 0.25 |
| HDL cholesterol (mg/dl) | 0.95 |
| LDL cholesterol (mg/dl) | 0.43 |
| RLP cholesterol (mg/dl) | 0.58 |
| Apo A-I (mg/dl) | 0.85 |
| Apo B (mg/dl) | 1.00 |
| Apo B-48 (µg/ml) | **0.01** |
| Apo E (mg/dl) | 1.00 |
| Lathosterol (µg/ml) | 0.40 |
| Campesterol (µg/ml) | 0.99 |
| Sitosterol (µg/ml) | 0.88 |

Change in parameters between baseline and 24 weeks were also compared using a permutation test. HDL, high-density lipoprotein; LDL, low-density lipoprotein; RLP, remnant lipoprotein; Apo, apolipoprotein. P-values < 0.05 are shown in bold.
