# Supplementary material for: Dipeptidyl peptidase-4 inhibitor anagliptin reduces fasting apolipoprotein B-48 levels in patients with type 2 diabetes: A randomized controlled trial
Source: PLoS One. 2020 Jan 28;15(1):e0228004. doi: 10.1371/journal.pone.0228004 (PMC6986701; doi:10.1371/journal.pone.0228004)
Supplement: S2 Appendix — (PDF) [file pone.0228004.s004.pdf]

別記様式第２号の４

２型糖尿病患者の血中脂質に対するDPP4阻害薬スイニー（アナグリプチン）の効果に関する研究

研究計画書
（臨床介入研究）

研究責任者：
職名・氏名：名古屋大学大学院医学系研究科　糖尿病・内分泌内科学
教授・有馬　寛
住所：名古屋市昭和区鶴舞町65
直通電話番号：052-744-2191
FAX番号：052-744-2191
e-mail: arima105@med.nagoya-u.ac.jp
研究事務局：
機関名・所属部局名：名古屋大学大学院医学系研究科　糖尿病・内分泌内科学
職名・氏名：病院助教・尾上　剛史
住所：名古屋市昭和区鶴舞町65
直通電話番号：052-744-2142
FAX番号：052-744-2206
e-mail：t-onoue@med.nagoya-u.ac.jp

2014 年 2 月 25 日 初版承認
2018 年 10 月 18 日 作成 Ver.6
2018 年 12 月 12 日 承認 Ver.6

Ⅰ　課題名

和文：２型糖尿病患者の血中脂質に対するDPP4阻害薬スイニー（アナグリプチン）の効果に関する研究

英文：The effects of anaglipitin, a DPP-4 inhibitor, on blood lipids in type 2 diabetic patients.

（略称：Anagliptin effects on lipids、ANGELS）

Ⅱ　研究組織

１　研究責任者（所属・職名・氏名）

名古屋大学大学院医学系研究科　糖尿病・内分泌内科学

教授・有馬　寛

２　研究分担者（所属・職名・氏名）

名古屋大学大学院医学系研究科　糖尿病・内分泌内科学・講師・坂野　僚一

同・病院講師・後藤　資実

同・講師・須賀　英隆

同・助教・髙木　博史

同・病院助教　恒川　卓

同・病院助教　尾上　剛史

同・大学院生　古川　麻里子

同・大学院生　和田　絵梨

名古屋大学大学院医学系研究科　CKD 先端医療システム寄附講座

助教・伊藤　禎浩

名古屋大学医学部附属病院　先端医療・臨床研究支援センター・准教授・安藤　昌彦

３　共同研究者（所属・職名・氏名）

海南病院糖尿病内分泌科・部長・山守越子

４　効果安全性評価委員会

・委員の所属・職名・氏名

藤田保健衛生大学生理学Ⅰ・教授・長崎弘

大垣市民病院糖尿病腎臓内科　藤谷　淳

名古屋大学大学院医学系研究科糖尿病内分泌内科学・大学院生　細川　香里

名古屋大学大学院医学系研究科糖尿病内分泌内科学・大学院生　村瀬　正敏

・研究実施モニタリング間隔

6か月

Ⅲ　研究等の概要

<研究の目的・意義・背景>

糖尿病治療においては、血糖値のみならず、血圧、脂質、肥満の改善が重要である。ジペプチジルペプチダーゼ-4（DPP4）阻害薬は、近年その使用頻度が急速に増加している血糖降下薬であるが、血糖改善効果のみならず、肥満抑制、血圧低下、膵β細胞保護など、糖尿病治療において多くの好ましい作用を有することが報告されている。2012年に承認された新しいDPP4阻害薬であるスイニー（一般名アナグリプチン）は、第３相試験において、上記に加えて血清コレステロールおよび中性脂肪の改善効果が観察されている。しかし脂質分画に対する効果および脂質改善作用の機序についての検討はなされていない。

本研究は、２型糖尿病患者において、アナグリプチンの脂質改善効果を検討する目的で施行する、無作為割り付け前向き臨床試験である。

脂質異常症は糖尿病に伴う大血管障害の危険因子として極めて重要であるが、既存の脂質改善薬を用いても良好なコントロールが得られない症例も多い。糖尿病患者におけるDPP4阻害薬の脂質改善効果の詳細が明らかとなれば、大血管障害の予防において大きな臨床的意味を持つ。またその機序の解明は、脂質異常症の新たな治療法の開発につながる可能性がある。これらの理由から本研究は有意義かつ必要である。

<研究の対象>

１　選択基準

・２型糖尿病患者
・HbA1c（NGSP）6.5 % 以上
・血清LDLコレステロール値　120 mg/dl以上
・過去3ヵ月以上にわたり、DPP4阻害薬による治療を受けていない。
・過去3ヵ月以上にわたり、内服薬なし、もしくは内服薬*の変更を受けていない。

＊メトフォルミン、α－ＧＩ、チアゾリジン、グリニドは、量・組み合わせに関わらず併用可。低用量のスルフォニル尿素薬（グリメピリド 2mg/日以下、ｸﾞﾘｸﾗｼﾞﾄﾞ 40mg/日以下、グリベンクラミド1.25m/日以下）および40単位／日以下のインスリン製剤の併用も可。

・過去3ヵ月間のHbA1cの変化量（絶対値）が2%未満である。
・年齢２０歳以上
・同意能力を有しており、本人から文書同意の取得が可能な患者

２　除外基準

・1 型糖尿病

・高用量のスルフォニル尿素薬（グリメピリド 2mg/日超、ｸﾞﾘｸﾗｼﾞﾄﾞ 40mg/日超、グリベンクラミド 1.25m/日超）および高用量のインスリン製剤（40 単位／日超）を使用中の患者。

・重症低血糖または無自覚性低血糖を繰り返し発現する患者。

・重篤な肝疾患（例えば非代償性肝硬変）を有する患者、あるいは前治療期の検査で AST（GOT）または ALT（GPT）が 100IU/L 以上の患者。

・透析中の患者

・重篤な心疾患を有する患者、あるいは前治療期開始前 6 カ月以内に心筋梗塞を起こした患者。

・高度の貧血(Hb 8.0 g/dl 未満)の患者

・重篤な膵疾患（例えば急性膵炎）を有する患者。

・悪性腫瘍を有する患者。

・治療不十分な内分泌疾患（下垂体・副腎・甲状腺疾患など）を有する患者。

・重症感染症、手術前後、重篤な外傷のある患者。

・高度な糖尿病性神経障害を有する患者。

・光凝固または硝子体手術が必要な糖尿病網膜症を有する患者。

・重症ケトーシス、糖尿病性昏睡または前昏睡の患者。

・炎症性腸疾患、大腸潰瘍、局所的腸閉塞、腸閉塞素因のある患者。

・消化管切除の既往のある患者。

・過度の常習飲酒者（例えば 1 日平均日本酒では 3 合、ビールでは大びん 3 本以上)。

・妊婦または妊娠している可能性のある女性、妊娠を希望している女性および授乳中の女性。

・アナグリプチンおよび DPP4 阻害薬に対して過敏症その他副作用の既往がある患者。

・その他、担当医師が不適当と判断した患者。

３　目標症例数

目標症例数：６０例

設定根拠

本研究を実施する 4 施設における症例集積は、年間 30 例程度と予想される。探索的な検討であることを考慮し、2 年間で集積可能な 60 例を目標症例数に設定した。

血清脂質の各指標について、登録時と登録 24 週後の測定値による前後差を算出する。この前後差について、治療群間での差を有意水準 0.05（両側）で検定した場合、一群 30 例、合計 60 例での検出力は以下の通りとなる。

・治療群間差/標準偏差が 65%の場合、検出力 71%
・治療群間差/標準偏差が 70%の場合、検出力 77%
・治療群間差/標準偏差が 75%の場合、検出力 83%
・治療群間差/標準偏差が 80%の場合、検出力 87%

４　採取する試料等

A. 人体から得られる試料

■血液材料

利用目的：含有物質（下記詳述）の測定

採血量：19 mL/例　　採血回数：2 回/例

採血方法：一般検体採血時に追加する。

侵襲性の有無とその理由：採血に伴う侵襲があるが、通常の採血時に合わせて血液を追加採取するため採血回数は増えず、追加血液量も少量であることから、通常採血に比べ侵襲の増大はほぼないと言える。

□病理材料（対象臓器名　　　　）

利用目的：

量・大きさ：　/例　　採取回数：　回/例

採取方法：

侵襲性の有無とその理由：

□生検材料（対象臓器名　　　　）

利用目的：

量・大きさ：　/例　　採取回数：　回/例

採取方法：

侵襲性の有無とその理由：

□細胞（採取部位　　　　　）

利用目的：

数量：　個/例　　採取回数：　回/例

採取方法：

侵襲性の有無とその理由：

□その他（　　　　　　　）

利用目的：

数量：　/例　　採取回数：　回/例

採取方法：

侵襲性の有無とその理由：

・上記材料の採取期間　　　　　2014 年 1 月　～　　2018 年 12 月
・試料の保管場所：名古屋大学大学院医学系研究科　代謝病態学寄附講座
・新規採取試料

B. 使用する診療情報等

■カルテから転記する項目

身長、体重、BMI、血圧、内服薬、糖尿病罹病歴

・上記診療情報等の収集期間　　2013 年 12 月　～　2018 年 12 月
・診療情報等の保管場所（名古屋大学大学院医学系研究科糖尿病・内分泌内科）
・新規収集診療情報等か、既存診療情報等か：新規収集診療情報等

＜研究期間＞

実施承認日～2020 年 3 月 31 日）

＜研究方法＞

１　試験デザイン：無作為割り付け前向き臨床試験（多施設共同試験）

２　試験方法：無作為割り付け後、以下の方法で投与を行う。投与期間は 24 週間とする。

A. スイニー群：スイニー1 回 100 mg を 1 日 2 回経口投与。効果不十分の場合には 1 回 200mg を 1 日 2 回まで増量可能とする。ただし、血清クレアチニン >2.4mg/dl（男性）、>2.0 mg/dl（女性）の場合は 1 日 1 回 100mg（効果不十分の場合には 1 回 100mg を 1 日 2 回まで増量可能）とする。併用薬の変更（用量変更も含む）は禁止とするが、低血糖出現のためやむをえない場合は減量を認める。

B. 対照群：対照群は試験期間中、原則として開始時点の血糖降下薬を継続するが、必要に応じて増量もしくは DPP4 阻害薬以外の新規薬剤を追加する。

スケジュールは下記の通りである。

時期	前治療期		被験薬投与期間						
	①	②	0 週	4 週	8 週	12 週	16 週	20 週	24 週
来院	◎	◎	◎			◎			◎
同意説明	◎								

適格性検査		◎							
同意書取得		◎							
空腹時採血*			◎						◎
体重、血圧			◎			◎			◎
自他覚随伴症状			◎			◎			◎

*：前治療期は投与開始前４ヶ月以内とする。

*：空腹時採血項目は、７．検査項目、検査時期の項参照。

３　割付：中央割り付け（Web による登録）

文書同意取得後、Web システムにて無作為割付を行う。登録された被験者はランダムに A 群（スイニー群）と B 群（対照群）に割り付けられる。

割付け方法は、下記の因子を割付調整因子とする最小化法を用いる。

・施設
・年齢（55 歳未満/55 歳以上）
・性別

登録割付責任者

安藤昌彦
名古屋大学医学部附属病院先端医療・臨床研究支援センター
TEL 052-744-1957
mando@med.nagoya-u.ac.jp

４　主要評価項目、副次的評価項目

A　主要評価項目

・血中脂質（下記項目）

B　副次的評価項目

・HbA1c、炎症性マーカー、酸化ストレスマーカー、サイトカイン（下記項目）

５　検査項目、検査時期

A　空腹時採血検査（2mlx2、6mlx1 本、9mlx1 本、および一般採血）：

０、２４週

１）脂質：TG, TC, HDL-C, LDL-C, RLP-C, ApoA1, ApoB48, ApoB100, ApoE
シトステロール、カンペステロール、ラソステロール
Lecithin-cholesterol acyltransferase (LCAT)、
Cholesteryl ester transfer protein (CETP)、
Proprotein convertase subtilisin/kexin type 9 (PCSK9)

(CETP、PCSK9 は名大研究室にて測定)

２）血糖関連：血漿グルコース、HbA1c

３）血漿インスリン濃度

４）サイトカイン：　アディポネクチン、TNF－α、MCP-1

５）酸化ストレスマーカー：MDA-LDL

６）炎症マーカー：hsCRP

７）一般採血：血球計算、肝・腎機能

B　体重、BMI、血圧：０，１２，２４週

６　統計学的手法

主要評価項目である血清脂質の各指標について、治療群ならびに登録時測定値を説明変数、登録時と登録 24 週後の測定値による前後差を結果変数とする共分散分析を用いて治療群間で比較する。なお、本試験において治療群・血清脂質測定値と有意に関連する因子が見い出された場合には新たに説明変数へ追加する。探索的な検討であるため、有意水準は常に両側 0.05 とし、検定の多重性に関する調整は行わない。

副次的評価項目である HbA1c、炎症性マーカー、酸化ストレスマーカー、サイトカイン等について、治療群ならびに登録時測定値を説明変数、登録時と登録 24 週後の測定値による前後差を結果変数とする共分散分析を用いて治療群間で比較する。なお、本試験において治療群・各指標測定値と有意に関連する因子が見い出された場合には新たに説明変数へ追加する。有意水準は両側 0.05 とし、検定の多重性に関する調整は行わない。

体重・BMI・血圧について、治療群を固定効果、登録時と登録後 12・24 週時点における測定値の前後差を結果変数とする線形混合モデルを用いて治療群間で比較する。共変量として登録時測定値、測定時点、治療群と測定時点の交互作用項を用いる。なお、本試験において治療群・各指標測定値と有意に関連する因子が見い出された場合には新たに説明変数へ追加する。有意水準は両側 0.05 とし、検定の多重性に関する調整は行わない。

７　多施設共同研究における本学の役割

本学が研究を主導し、各施設の検査結果をとりまとめて解析した後、論文等で発表する。

＜使用する研究費＞

■受託研究費（三和化学研究所、「2型糖尿病患者の血中脂質に対するDPP4 阻害薬スイニー(アナグリプチン)の効果の検討」)

＜共同研究機関＞
共同研究機関の名称、共同研究機関での倫理審査体制
　　海南病院糖尿病内分泌科：IRB にて審査

本研究における本学と共同研究機関のそれぞれの役割
　　各施設で得た検体・患者情報を本学に集めて解析する。

＜外部委託＞
株式会社 SRL に大部分の測定を依頼する。依頼には試験専用の ID 番号を用いる。

Ⅳ　研究の実施場所
名古屋大学大学院医学系研究科　糖尿病・内分泌内科
海南病院糖尿病内分泌科

Ｖ　実施に際しての倫理的配慮について
Ｖ―１＜インフォームド・コンセントについて＞
説明者の氏名
　尾上剛史
説明の方法
■文書を用いる。
□説明の内容と同意の記録を作成する。
□同意を取得しない。
　　理由：

説明書・同意書の保管場所及び保管方法
　収集した説明書・同意書は代謝病態学講座で施錠可能なロッカーに保管する。
研究実施についての情報公開の有無
　情報公開しない。

Ｖ―２＜個人情報の取り扱いについて＞
１　個人情報保護の具体的方法
　各患者に試験専用 ID 番号を割り当て、連結表を作成のうえ、氏名、イニシャル、カルテ ID などの個人情報を削除して連結可能匿名化する。個人情報管理者が連結表などを施錠可能なロッカーに保管する。データを保管するパソコンやＵＳＢにはパスワードロックをかけ、連結表とは別の施錠可能なロッ

カーに保管する。

２　個人情報管理者が必要な場合
　個人情報管理者
　　氏名：尾上剛史
　　資格：医師
　個人情報管理補助者
　　氏名：和田絵梨
　　資格：医師

Ｖ－３<同意の撤回>
　撤回は患者の自由意思でいつでも可能であり、それに伴う不利益は一切生じないことを説明書に明記する。同意を撤回した場合は試料を廃棄する。

Ｖ―４<対象者が未成年又は成人でも十分な判断力がないと考えられる場合への対処方法>
■A　下記特例を対象にしない。
□B　未成年者
□C　十分な判断力がない成年者
□D　意識のない成年者
□E　病名に対する配慮が必要な成年者
□F　その他（　　　　　　　　　）

Ｖ―５<分析結果の開示>
　個人の検査結果については原則本人に開示する。
Ｖ－６<謝礼・手当>
　支給しない。
Ｖ－７<費用負担>
　研究目的で実施する検査に必要な費用は、担当講座が支払い患者負担は一切生じない。その他は通常の医療費となる。

Ⅵ　既存試料の利用

１　既存試料の利用　□あり　■なし

Ⅶ　期待される研究成果あるいは予測される利益

期待される研究成果：

DPP4 阻害薬による脂質改善効果およびその機序が明らかになれば、糖尿病合併症（特に大血管障害）発症進行予防のための薬剤選択根拠に影響を与えるとともに、今後の脂質改善薬の開発において大きな意義がある。

被験者が得られると期待される利益について：

合併症防止のため、より適切な薬剤の選択肢が増えるとともに、脂質の改善が得られる、もしくは脂質低下薬を減量・中止できる可能性が生じる。

Ⅷ　予測される危険と不利益とそれに対する配慮・補償

Ⅷ－１＜研究等によって対象者に生じうる危険と不快に対する具体的配慮＞

スイニーおよび対照群で使用される可能性のある薬剤はいずれも承認済みで通常の用量・用法を遵守するため、基本的な安全性は証明されている。以下の危険・不利益の可能性がある。

１）　スイニーで報告されている主な副作用：低血糖症、消化器症状（便秘、下痢、腹部不快感など）、など。発生した場合には投薬・試験を中止し、適切な医療行為を施行する。

２）　採血時の痛み、出血、神経損傷など：試験用の採血は一般採血時に追加で行うため、採血針を刺す回数は増えない。従って採血時の痛み・神経損傷などの危険性の増大はないと考えられる。また採血量は一般採血を含め１回 26 ml、24 週で計 52 ml であり、これに伴う副作用はないと予想される。万一、不利益が発生した場合には最善の医療措置にて対応する。

Ⅷ－２＜対象者に健康被害が生じた場合の補償の有無及び具体的な措置＞

１　研究の種類

■介入を伴う研究であって、医薬品又は医療機器を用いた予防、診断又は治療方法に関するもの（対外診断を目的とした研究を除く。）

発生した健康障害に対しては、最善の医療措置により対応する。

本研究は薬剤による介入試験であるが、使用する薬剤はすべて承認済みで適正な用量・用法を遵守する内容であり、用量変更や適応拡大を目的としたものではない。従って、薬剤に起因する重篤な健康被害が生じた場合は、「医薬品副作用被害救済制度（医薬品医療機器総合機構）」の利用を考慮する。

また検査は通常採血時に少量の血液を追加採取するもので、侵襲・危険性が

増すことはほとんどない。万一採血時の神経損傷など健康被害が生じた場合は、最善の医療措置により対応する。

２　補償の種類

□補償のための保険を設定した。

□補償のための保険を設定する予定である。

■保険以外の対処方法を講じた。

□補償がない旨を説明する。

３　措置内容

■説明同意文書に補償のための保険等必要な措置について記載した。

（医薬品副作用被害救済制度）

□補償のための保険等必要な措置を証明する文書を添付した。

４有害事象が発生した場合の対応

　薬剤の副作用が発生した場合には投薬・試験を中止し、必要に応じて投薬、点滴など適切な医療行為を施行する。採血時の有害事象についても最善の医療措置を行う。

Ⅸ　研究終了後の試料等の取扱い

■　廃棄する。

廃棄の方法：通常の感染性廃棄物として処理業者に委託する。

□　保存する。

X　モニタリング・監査

　直接閲覧を伴うモニタリング、監査等の調査は行わない。

ＸＩ　利益相反

　本研究開始時の研究分担者２名は、本研究で使用する薬剤スイニーの製造販売企業である三和化学研究所からの寄附により設立された代謝病態学寄附講座の教員であった。また本研究の研究分担者１名は三和化学研究所からの寄付を含むＣＫＤ先進診療システム学寄附講座所属である。また同企業と糖尿病・内分泌内科が本研究に関して受託研究契約を結んでおり、利益相反の状態にある。

　しかしながら、本研究は医師主導により科学的根拠に基づき計画し、その実施、解析を行うので三和化学研究所は一切関与しない。また、症例の割り付け・

解析は当院の先端医療・臨床研究支援センターに依頼するので、試験を担当する医師は関与しない。これにより、研究の客観性を担保し、実施前にはUMINに登録し、いかなる結果であっても公表する予定である。これらの独立した体制により、企業に恣意的な有利な結果を排除し研究成果の公正性を保つ。

ＸⅡ　参考文献

(1) Kato N, Oka M, Murase T, Yoshida M, Sakairi M, Yamashita S, Yasuda Y, Yoshikawa A, Hayashi Y, Makino M, Takeda M, Mirensha Y, Kakigami T. Discovery and pharmacological characterization of N-[2-({2-[(2S)-2-cyanopyrrolidin-1-yl]-2-oxoethyl}amino)-2-methylpropyl]-2-methyl pyrazolo[1,5-a]pyrimidine-6-carboxamide hydrochloride (anagliptin hydrochloride salt) as a potent and selective DPP-IV inhibitor. Bioorg Med Chem. 2011 Dec 1;19(23):7221-7.

(2) 角南由紀子, 瀬底正吾, 加来浩平. 新規ジペプチジルペプチダーゼ-4阻害薬Anagliptinの単回および反復投与における薬物動態学的および薬力学的検討. Jpn Pharmacol Ther. 2012;40(10):847-58

(3) 加来浩平. 日本人2型糖尿病患者におけるAnagliptinの用量探索試験—多施設共同, ランダム化, プラセボ対照二重盲検群間比較試験—Jpn Pharmacol Ther. 2012;40(11):973-84.

(4) 加来浩平. 日本人2型糖尿病患者におけるAnagliptinの有効性および安全性の検討—多施設共同, ランダム化, プラセボおよび実薬対照二重盲検群間比較試験—Jpn Pharmacol Ther. 2012;40(11):985-95.

(5) 加来浩平. 日本人2型糖尿病患者に対するanagliptinの長期単独療法における有効性および安全性—多施設共同, ランダム化, オープンラベル, 並行群間比較試験（食前投与と食後投与の比較）—Jpn Pharmacol Ther. 2012;40(9):733-44.

(6) 加来浩平. 日本人2型糖尿病患者に対するanagliptinの併用療法における有効性および安全性—プラセボ対照, ランダム化, ダブルブラインド, 並行群間比較およびオープンラベル長期投与試験—Jpn Pharmacol Ther. 2012;40(9):745-70.

(7) 内野泰, 加来浩平. 新規ジペプチジルペプチダーゼ-4阻害薬 Anagliptinによる1日の血糖推移の改善効果 Jpn Pharmacol Ther. 2012;40(10):859-69.

(8) 金慧徳, 加来浩平. 新規DPP-4阻害薬Anagliptinとα-グルコシダーゼ阻害薬Miglitolの日本人2型糖尿病患者における薬物相互作用 Jpn Pharmacol Ther. 2012;40(10):871-81.

(9) 金慧徳, 加来浩平 新規DPP-4阻害薬AnagliptinとMetforminの日本人2型糖尿病患者における薬物相互作用 Jpn Pharmacol Ther. 2012;40(10):883-94.

(10) 加来浩平. 日本人 2 型糖尿病患者に対する anagliptin の血清脂質に及ぼす影響—Anagliptin 長期投与試験に関する併合解析—Jpn Pharmacol Ther. 2012;40(9):771-84.
(11) Ervinna N, Mita T, Yasunari E, Azuma K, Tanaka R, Fujimura S, Sukmawati D, Nomiyama T, Kanazawa A, Kawamori R, Fujitani Y, Watada H. Anagliptin, a DPP-4 Inhibitor, Suppresses Proliferation of Vascular Smooth Muscles and Monocyte Inflammatory Reaction and Attenuates Atherosclerosis in Male apo E-Deficient Mice. Endocrinology. 2013 Mar;154(3):1260-70.
(12) Nakaya K, Kubota N, Takamoto I, Kubota T, Katsuyama H, Sato H, Tokuyama K, Hashimoto S, Goto M, Jomori T, Ueki K, Kadowaki T. Dipeptidyl peptidase-4 inhibitor anagliptin ameliorates diabetes in mice with haploinsufficiency of glucokinase on a high-fat diet. Metabolism. 2013 Jul;62(7):939-51.
