# Supplementary material for: Dipeptidyl peptidase-4 inhibitor anagliptin reduces fasting apolipoprotein B-48 levels in patients with type 2 diabetes: A randomized controlled trial
Source: PLoS One. 2020 Jan 28;15(1):e0228004. doi: 10.1371/journal.pone.0228004 (PMC6986701; doi:10.1371/journal.pone.0228004)
Supplement: S3 Appendix — (PDF) [file pone.0228004.s005.pdf]

Effects of anagliptin, a DPP-4 inhibitor, on blood lipids in type 2 diabetic patients

Study Protocol (Translation)

(Interventional study)

Principal Investigator: Hiroshi Arima, M.D., Ph.D.
Nagoya University Graduate School of Medicine, Department of Diabetes and Endocrinology
Address: 65 Tsurumai-cho, Showa-ku, Nagoya City

Research Office: Takeshi Onoue, M.D., Ph.D.
Nagoya University Graduate School of Medicine, Department of Diabetes and Endocrinology
Address: 65 Tsurumai-cho, Showa-ku, Nagoya City
Phone number: 052-744-2142
Fax number: 052-744-2206

Approved Date (original ver.): February 25, 2014
Updated Date (ver. 6): October 18, 2018
Approved Date (ver. 6): November 12, 2018

I Study name

Effects of anagliptin, a DPP-4 inhibitor, on blood lipids in type 2 diabetic patients (Anagliptin effects on lipids, ANGELS)

II Research organization

1. Principal Investigator:

Nagoya University Graduate School of Medicine, Department of Diabetes and Endocrinology

Professor Hiroshi Arima

2. Subinvestigator:

Nagoya University Graduate School of Medicine, Department of Diabetes and Endocrinology

Ryoichi Banno
Motomitsu Goto
Hidetaka Suga
Hiroshi Takagi
Taku Tsunekawa
Takeshi Onoue
Mariko Furukawa
Eri Wada
Yasuhiro Ito

Center for Advanced Medicine and Clinical Research, Nagoya University Hospital

Masahiko Ando

3. Co-researcher:

Department of Endocrinology, Department of Diabetes, Kainan Hospital, Aichi Prefectural Welfare Federation of Agricultural Cooperatives

Etsuko Yamamori

4. Data and Safety Monitoring Board:

Professor Hiroshi Nagasaki, Fujita Medical University
Atushi Fujiya, Ogaki Municipal Hospital
Kori Hosokawa, Nagoya University Graduate School of Medicine, Department of Diabetes and Endocrinology
Masatoshi Murase, Nagoya University Graduate School of Medicine, Department of

Diabetes and Endocrinology

・Monitoring interval: 6 months

III Background

In the treatment of diabetes, improvements in blood pressure, lipids, and obesity are important in addition to improvements in blood sugar level. Dipeptidyl peptidase-4 (DPP-4) inhibitors are oral hypoglycemic agents that have been widely used in the treatment of type 2 diabetes. In addition to their hypoglycemic effects, DPP-4 inhibitors have protective effects on pancreatic β-cells and anti-atherogenic effects, renoprotective effects, and favorable effects on lipid metabolism.

Anagliptin is a DPP-4 inhibitor that was launched in 2012 and reportedly lowered LDL cholesterol levels in a long-term phase III trial. Since then, several studies have confirmed its beneficial effect on lipid metabolism by showing that the drug decreases serum cholesterol and triglyceride levels in type 2 diabetics. However, the effects of anagliptin on lipid profiles have not fully been investigated.

The present study is a randomized and prospective clinical trial, which investigates the lipid-improving effects of anagliptin in patients with type 2 diabetes.

Dyslipidemia is an extremely important risk factor for macroangiopathy associated with diabetes. The findings of this study will expand our understanding of how anagliptin affects lipid metabolism and also provide insights into the molecular mechanism of its action.

<Patients>

1. Inclusion criteria:

1) Type 2 diabetes
2) Glycosylated hemoglobin (HbA1c) ≥ 6.5%
3) Serum LDL cholesterol ≥ 120 mg/dl
4) Not been treated with DPP-4 inhibitors for the past 3 months
5) No antidiabetic medication or did not change their medication for the past 3 months
   *Metformin, α-GI, thiazolidine, and glinide can be used together regardless of the amount and combination. Low-dose sulfonylurea drug (glimepiride 2 mg/day or less, gliclazide 40 mg/day or less, glibenclamide 1.25 mg/day or less) and insulin 40 units/day or less can be used together.
6) Less than 2% HbA1c change (absolute value) for the past 3 months
7) 20 years of age or older

2. Exclusion criteria:

1) Type 1 diabetes
2) Treated with insulin, glinides, or high-dose sulfonylureas (glimepiride > 2 mg/day, gliclazide > 40 mg/day, glibenclamide > 1.25 mg/day)
3) Hypoglycemia unawareness or recurrent severe hypoglycemia
4) Severe forms of liver disease (aspartate transaminase or alanine aminotransferase ≥ 100 IU/L)
5) On dialysis
6) Severe forms of cardiac disease
7) Severe anemia (Hb < 8.0 g/dl)
8) Severe pancreatitis
9) Any malignant diseases
10) Uncontrolled endocrine diseases
11) Severe infections or were undergoing surgical treatment
12) Severe diabetic complications
13) Inflammatory bowel disease, colon ulcers, ileus, or a history of intestinal resection
14) Addiction to alcohol
15) Pregnant or possibly pregnant
16) Allergy to DPP-4 inhibitors
17) Assessed by their physicians to be unsuitable for participation in the study

3. Number of patients:

60 patients

We expect 30 cases from participating hospitals annually. In consideration of this exploratory study, we set the target number as 60 cases accumulated over 2 years. Changes in parameters between baseline and 24 weeks are evaluated. When the difference between treatment groups is tested at a significance level of 0.05 (two sided), the power of 30 cases in a total of 60 cases is as follows:

・ When the difference between treatment groups/standard deviation is 65%, the statistical power is 71%.

・ When the difference between treatment groups/standard deviation is 70%, the statistical power is 77%.

・ When the difference between treatment groups/standard deviation is 75%, the statistical power is 83%.

・ When the difference between treatment groups/standard deviation is 80%, the

statistical power is 87%.

4. Samples:

A. Samples obtained from the human body

■ Blood sampling

Purpose of use: Measurement of the following items

Blood sampling volume: 19 mL

Frequency: twice/patient

Method: Add at the time of usual blood test

Presence or absence of invasiveness and reasons: Although a blood test is somewhat invasive, samples are collected in conjunction with routine clinical blood tests, and this additional blood volume is small.

Period: From January 2014 to December 2018

Storage place: Nagoya University Graduate School of Medicine

B. Information

■ Items collected from medical records

Height, body weight, body mass index (BMI), blood pressure, medication, and history of diabetes

Period: From December 2013 to December 2018

Storage place: Nagoya University Graduate School of Medicine

<Study period>

From approval date to March 31, 2020

<Method>

1. Study design: Randomized prospective clinical trial (multicenter collaborative study)
2. Method: After random assignment, administration will be performed as follows. The administration period is 24 weeks.

Anagliptin group: Patients in the anagliptin group are treated with 200 mg of the drug twice daily for 24 weeks. However, if serum creatinine > 2.4 mg/dl (male) or > 2.0 mg/dl (female), patients will be treated with 100 mg once daily. No medications will be added

throughout the study. Drugs currently taken by patients at the time of enrollment will continue to be administered.
Control group: Patients in the control group did not receive anagliptin but continue with their previous treatment schedules.

The schedule is as follows.

	Pre-intervention period		Intervention period (week)						
	(1)	(2)	0	4	8	12	16	20	24
Visit	*	*	*			*			*
Informed consent	*								
Eligibility check		*							
Obtain consent form		*							
Blood test (fasting)			*						*
Weight, blood pressure			*			*			*
Symptoms			*			*			*

Pre-intervention period is within 4 months before intervention starts.

3. Assignment: Central assignment (web-based registration)
After consent is obtained from the patients, the researcher accesses a web-based registration system developed by the Center for Advanced Medical and Clinical Research at Nagoya University Hospital and enters the information required for enrollment. The system automatically determines the eligibility of each patient and randomly assigns him/her to the anagliptin (n = 12) or control (n = 12) group with a dynamic allocation strategy using a minimization method. Stratification includes the hospital that the patient visited, sex, and age (>55 or ≤55).

Person in charge of registration assignment

Masahiko Ando

Center for Advanced Medicine and Clinical Research, Nagoya University Hospital

Telephone: 052-744-1957

4. Outcomes
A Primary outcome:

Serum lipid profiles

B Secondary outcomes:

HbA1c, cytokines, oxidative stress markers, and inflammation markers

5. Laboratory data

A Laboratory data will be measured under fasting conditions at baseline and 24 weeks (2 ml × 2, 6 ml × 1 bottle, 9 ml × 1 bottle, and usual blood test).

1) Serum lipid profiles: TG, TC, HDL-C, LDL-C, RLP-C, ApoA1, ApoB48, ApoB100, ApoE, Sitosterol, Campesterol, Lanosterol, lecithin-cholesterol acyltransferase, cholesteryl ester transfer protein (CETP), proprotein convertase subtilisin/kexin type 9 (PCSK9) (CETP and PCSK9 will be measured at Nagoya University)
2) Glycometabolism: HbA1c and fasting plasma glucose
3) Blood insulin
4) Cytokine levels: serum adiponectin, serum tumor necrosis factor α, and serum monocyte chemoattractant protein-1
5) Oxidative stress marker: serum malondialdehyde-modified LDL
6) Inflammation marker: serum high-sensitivity C-reactive protein
7) Usual blood test: blood count, liver function, and kidney function

B Body weight, BMI, and blood pressure will be measured at 0, 12, and 24 weeks.

6. Statistical analysis

Changes in serum lipid profiles (primary outcomes) between baseline and 24 weeks are evaluated in each group and compared using an analysis of covariance (ANCOVA) model with the baseline value and treatment group as covariates. Since this work is an exploratory study, no adjustment for multiplicity is performed, and analyses are conducted using two-sided statistical tests at a significance level of 0.05.

Changes in glycometabolism, cytokine levels, oxidative stress marker levels, and inflammation marker levels between baseline and 24 weeks are evaluated in each group and compared using ANCOVA model with the baseline value and treatment group as covariates. No adjustment for multiplicity is performed and analyses are conducted using two-sided tests at a significance level of 0.05.

Changes in body weight, BMI, and blood pressure are evaluated in each group and compared using a liner mixed model in which the treatment variables are the fixed effects and differences between measured values at enrollment and at 12 and 24 weeks

after registration. We use baseline value, measurement time, interaction of treatment group, and measurement time as covariates. No adjustment for multiplicity is carried out and analyses are conducted using two-sided tests at a significance level of 0.05.

7. Role of Nagoya University

Researchers at Nagoya University will lead the research by collecting and analyzing samples and patient information of each hospital. The results will be presented as a research manuscript.

<Research expenses>

This study was funded by Sanwa Kagaku Co., Ltd.

<Joint research institute>

Name of joint research institute, ethics review system in joint research institute

Kainan Hospital, Department of Diabetes and Endocrinology: reviewed by IRB

Roles of Nagoya University and joint research institute in this study

Researchers at Nagoya University will lead the research by collecting and analyzing samples and patient information of each hospital. The results will be presented as a research manuscript.

<Outsourcing>

Measurements of serum lipid profiles and other parameters are outsourced to a commercial clinical testing laboratory (SRL, Tokyo, Japan). Samples are measured anonymously using a subject identification code.

IV Study site

Nagoya University Graduate School of Medicine

Kainan Hospital, Department of Diabetes and Endocrinology

V Ethical considerations

V-1 <Informed Consent>

Name of the presenter

Takeshi Onoue

Method of explanation

■ Use documents (Informed Consent Form [ICF]).

□ Make records of the consent.

□ Do not get consent.

The reason:

Storage place and storage method of ICF:

The collected ICFs will be stored under lock at Nagoya University.

Existence of information disclosure about research:

Do not disclose information.

V-2 <Handling of Personal Information>

1. Method of personal information protection:

We assign a subject identification code to each patient, create a consolidated table, and make it connectable but anonymous. A personal information manager keeps this consolidated table under lock. The personal computer and HDD that store the anonymized data are secured with a password and kept locked separately from the consolidated table.

2. Personal information of manager:

Name: Takeshi Onoue

Qualification: Doctor

Personal information of management assistant:

Name: Eri Wada

Qualification: Doctor

V-3 <Withdrawal of Consent>

The ICF clearly states that withdrawal is always possible, and no disadvantages will be associated with it. If consent of the patient is withdrawn, the sample is discarded.

V-4 <Participants Who Are Juveniles or Adults without Sufficient Judgment>

■ The following persons are not eligible:

□ A Juveniles

□ B Adults without sufficient judgment

□ C Unconscious adult

□ D Adults who are not informed of the name of his/her disease

□ E other ( )

V-5 <Disclosure of Analysis Results>

Individual results will be disclosed to the individual.

V-6 <Reward and Allowance>

Not paid

V-7 <Patient cost>

The cost for examinations conducted for research purposes is paid by hospitals. The costs for other treatments are as usual.

VI Use of existing samples

1. Use of existing samples □ Yes ■ No

VII Expected research results or expected benefits

Expected research results:

These findings will help increase our understanding of the effects of anagliptin treatment on lipid metabolism and can offer suggestions as to the molecular mechanism of its action.

Expected benefits for patients:

Patients may see an improvement in lipid profile and thus might stop or reduce their lipid-lowering drugs.

VIII Predicted risks and disadvantages

VIII-1 <Considerations for Potential Risks and Disadvantages in Patients>

Suiny and the drugs that may be used in the control groups have been proven to be safe, as they are administered in the usual doses and regimens that have been previously approved.

The risks and disadvantages are as follows:

1) Side effects reported in Suiny:
   Hypoglycemia, digestive symptoms (constipation, diarrhea, abdominal discomfort, etc.), etc. If any side effects occur, patients will discontinue medication and are treated properly.
2) Pain, bleeding, nerve damage, etc., associated with blood test:

The blood tests are performed at the time of routine clinical blood sampling and thus does not increase the frequency of collection. The additional blood volume is small (26 mL per sample and 52 mL total during the study). If any adverse events occur, patients are properly treated.

VIII-2 <Compensation and Response when Adverse Events Occur in Patients>

1. Type of study

■ Interventional studies using drugs or medical devices

We provide the best treatment for adverse events caused by this study.
Although this study is an interventional study, all medications used are approved and administered properly. Therefore, if serious side effects resulting from medications occur, the "Pharmaceutical Side Effect Relief System (Pharmaceuticals and Medical Devices Agency)" is used.
In addition, blood tests are performed at the time of routine clinical blood sampling and thus does not increase the frequency of collection. The additional blood sample volume is very small. If any adverse events occur at the time of blood test, patients are properly treated.

2. Types of compensation

□ Insurance for compensation.
□ Insurance for compensation (planned).
■ Take measures other than insurance.
□ Explain that there is no compensation.

3. Measures

■ We describe compensation in the ICF.
(Pharmaceutical Side Effect Relief System)

4. Response to adverse events

If any side effects occur, patients will discontinue medication and are properly treated.
If any adverse events occur at the time of blood test, patients are properly treated.

IX Handling of samples after research

■ Disposal
Disposal method: Treat as normal infectious waste

□ Save

X Monitoring and audit

We do not conduct surveys, such as monitoring and audits, in this study.

XI Conflict of interest

At the start of this study, two research co-workers were faculty members of the Department of Metabolic Medicine, which was established by a donation from Sanwa Kagaku Co., Ltd., which is a company that manufactures and sells the medicine used in this study. In addition, one researcher in this study belongs to Department of Nephrology/CKD initiatives, which receives donations from Sanwa Kagaku Co., Ltd. Also, the company and Nagoya University have been contracted for this research.

However, this research is planned based on a scientific basis led independently by researchers, and subsequent implementation and analysis are also performed by researchers. Sponsors have no control over interpretation, writing, or publication of this work. In addition, since the assignment and analysis will be conducted by our advanced medical and clinical research support center, the doctor in charge of the study will not be involved.

This will ensure the objectivity of this study, which will be registered with UMIN prior to implementation. All results will be published. These independent systems ensure that the research results are fair and eliminating any biased results for the company.

XII References

(1) Kato N, Oka M, Murase T, Yoshida M, Sakairi M, Yamashita S, Yasuda Y, Yoshikawa A, Hayashi Y, Makino M, Takeda M, Mirensha Y, Kakigami T. Discovery and pharmacological characterization of N-[2-({2-[(2S)-2-cyanopyrrolidin-1-yl]-2-oxo ethyl}amino)-2-methylpropyl]-2-methyl pyrazolo[1,5-a]pyrimidine-6-carboxamide hydrochloride (anagliptin hydrochloride salt) as a potent and selective DPP-IV inhibitor. Bioorg Med Chem. 2011 Dec 1;19(23):7221-7.

(2) Sunami Y, et al. Pharmacokinetics and pharmacodynamics of single and multiple doses of anagliptin, a novel inhibitor of dipeptidyl peptidase-4. Jpn Pharmacol Ther. 2012;40(10):847-58

(3) Kaku K. Dose-ranging study of anagliptin in Japanese patients with type 2 diabetes -A multi-centre, randomized, placebo-controlled, double-blind,

parallel-group study-. Jpn Pharmacol Ther. 2012;40(11):973-84.
(4) Kaku K. Efficacy and safety of anagliptin in Japanese patients with type 2 diabetes -A multi-centre, randomized, placebo- and active comparator-controlled, double-blind, parallel-group study-. Jpn Pharmacol Ther. 2012;40(11):985-95.
(5) Kaku K. Efficacy and safety of long-term monotherapy with anagliptin in Japanese patients with type 2 diabetes -A multi-centre, randomized, open-label, parallel-group study (administered before meals vs. after meals). Jpn Pharmacol Ther. 2012;40(9):733-44.
(6) Kaku K. Efficacy and safety of anagliptin add-on therapy in Japanese patients with type 2 diabetes -Placebo-controlled, double-blind, parallel-group study with an open-label, long-term extension-. Jpn Pharmacol Ther. 2012;40(9):745-70.
(7) Uchino H, et al. A novel dipeptidyl peptidase-4 inhibitor, anagliptin, improved the daily blood glucose profile Jpn Pharmacol Ther. 2012;40(10):859-69.
(8) Kim H, et al. Drug interaction between anagliptin, a novel dipeptidyl peptidase-4 inhibitor, and miglitol, an α-glucosidase inhibitor, in Japanese patients with type 2 diabetes Jpn Pharmacol Ther. 2012;40(10):871-81.
(9) Kim H, et al. Drug interaction between anagliptin, a novel dipeptidyl peptidase-4 inhibitor, and metformin in Japanese patients with type 2 diabetes Jpn Pharmacol Ther. 2012;40(10):883-94.
(10) Kaku K. Effects of anagliptin on serum lipids in Japanese patients with type 2 diabetes -A pooled analysis of long-term therapy with anagliptin-. Jpn Pharmacol Ther. 2012;40(9):771-84.
(11) Ervinna N, Mita T, Yasunari E, Azuma K, Tanaka R, Fujimura S, Sukmawati D, Nomiyama T, Kanazawa A, Kawamori R, Fujitani Y, Watada H. Anagliptin, a DPP-4 Inhibitor, Suppresses Proliferation of Vascular Smooth Muscles and Monocyte Inflammatory Reaction and Attenuates Atherosclerosis in Male apo E-Deficient Mice. Endocrinology. 2013 Mar;154(3):1260-70.
(12) Nakaya K, Kubota N, Takamoto I, Kubota T, Katsuyama H, Sato H, Tokuyama K, Hashimoto S, Goto M, Jomori T, Ueki K, Kadowaki T. Dipeptidyl peptidase-4 inhibitor anagliptin ameliorates diabetes in mice with haploinsufficiency of glucokinase on a high-fat diet. Metabolism. 2013 Jul;62(7):939-51.
